# Determinants of exercise calf muscle perfusion in Peripheral Arterial Disease (PAD)

**DOI:** 10.1186/1532-429X-18-S1-P356

**Published:** 2016-01-27

**Authors:** Jorge A Gonzalez, Yan Li, Peter W Shaw, Jennifer Kay, Robyn McKenzie, David Lopez, Aditya Sharma, Joseph DiMaria, Yang Yang, Petroni Gina, Michael Salerno, Craig H Meyer, Frederick H Epstein, Brian H Annex, Christopher M Kramer

**Affiliations:** 1 grid.27755.32000000009136933XCardiology, University of Virginia, Charlottesville, VA USA; 2 grid.27755.32000000009136933XBiomedical Engineering, University of Virginia, Charlottesville, VA USA; 3 grid.27755.32000000009136933XRadiology, University of Virginia, Charlottesville, VA USA; 4 grid.27755.32000000009136933XDivision of Translational Research and Applied Statistics, University of Virginia, Charlottesville, VA USA

## Background

In patients with PAD, ankle-brachial index (ABI) does not correlate well with time to claudication and lacks the ability to quantify tissue perfusion, therefore limiting the development of new therapies. Arterial spin labeling (ASL) MRI is a novel non-contrast technique that measures peak calf muscle perfusion noninvasively at a microvascular level. We sought to analyze the relationship of traditional risk factors for PAD and levels of exercise-induced calf muscle perfusion measured by ASL MRI.

## Methods

Forty-two (42) patients with PAD (ABI < 0.9) were prospectively enrolled. All performed supine plantar flexion exercise using a pedal ergometer until exhaustion or limiting symptoms. Images of the most symptomatic leg were obtained at end exercise using a flexible calf coil in a 3-T Siemens Trio. Fifteen (15) averaged perfusion-weigthed ASL images were acquired over 1 minute post-exercise with single-shot echo-planar imaging readouts (field of view: 200 × 200 mm, matrix: 64 × 64, repetition time: 4,000 msec, echo time: 32 msec, slice thickness: 10 mm).

## Results

The mean age was 66 ± 11 years, 64% were male, 67% Caucasians and mean ABI was 0.60 ± 0.12.

Fifty-two (52%) were diabetics, 86% had hypertension, 79 % had hyperlipidemia (HLD), 62% had CAD, 91% were smokers and 14% had a prior TIA/Stroke. The mean BMI was 29.1 ± 5.0, mean GFR was 70.5 ± 23.7 mL/min/1.73 m^2^. Mean log(ASL) values were higher (1.39 ± 0.26) in patients with HLD than in those without HLD (1.17 ± 0.20) (R^2^= 0.12 p = 0.02). Other risk factors (HTN, smoking, gender and diabetes) did not correlate with ASL. Age is a significant predictor of log(ASL) (p < 0.05). For every year increase in age there is a 1.7% increase in ASL. ABI, however, was not associated with either hyperlipidemia or age.

## Conclusions

Tissue microvascular perfusion in PAD as measured by ASL has different determinants than the macrovascular disease measured by ABI. Older PAD patients with HLD may primarily suffer from the macrovascular aspects of the disease, rather than the microvascular.

**Figure 1:**
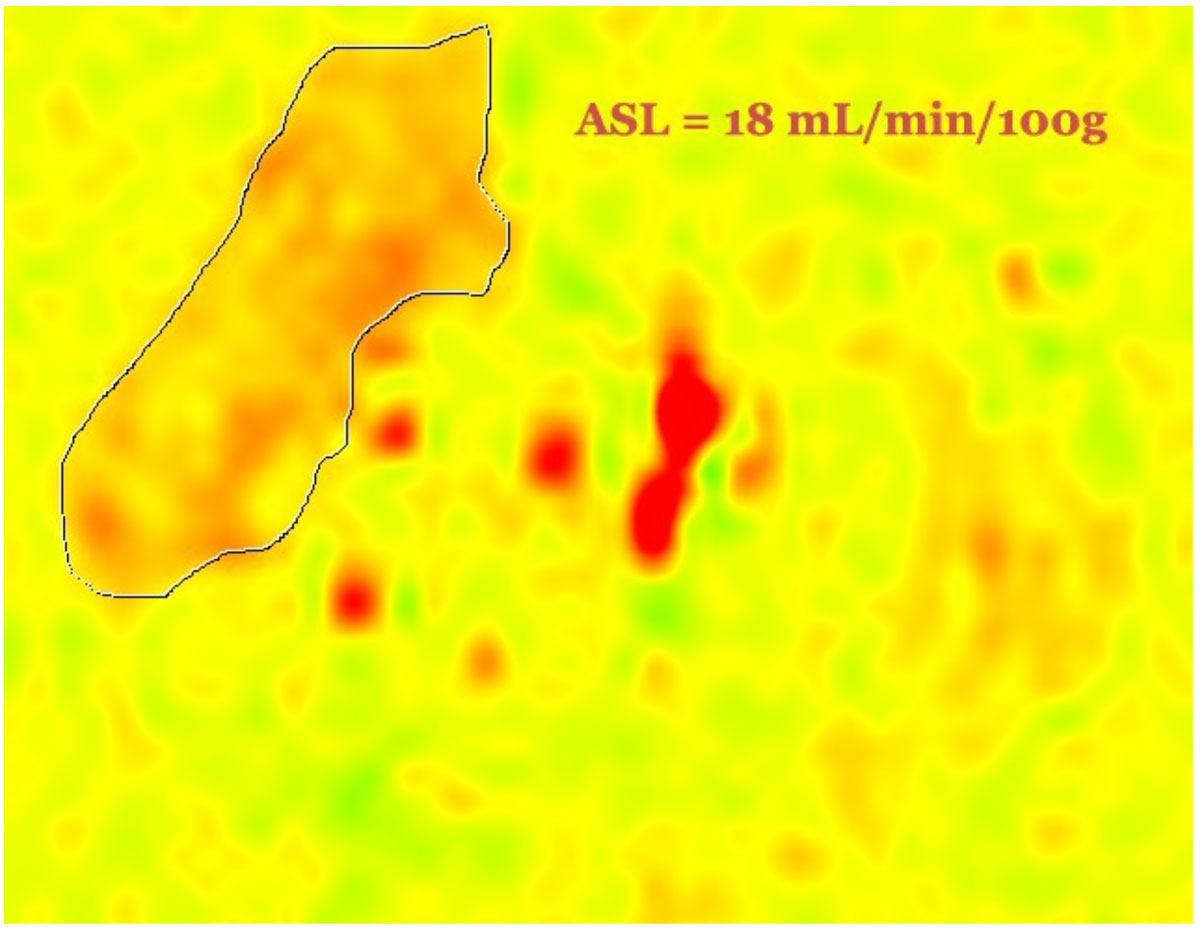
**ASL value calculated from ROI in the lateral compartment of the R leg of patient with PAD at peak exercise**.

